# Acquired rifampicin resistance during first TB treatment: magnitude, relative importance, risk factors and keys to control in low-income settings

**DOI:** 10.1093/jacamr/dlac037

**Published:** 2022-04-09

**Authors:** Armand Van Deun, Valentin Bola, Rossin Lebeke, Michel Kaswa, Mohamed Anwar Hossain, Mourad Gumusboga, Gabriela Torrea, Bouke Catharine De Jong, Leen Rigouts, Tom Decroo

**Affiliations:** 1 Independent Consultant, 3000 Leuven, Belgium; 2 Programme National de Lutte contre la Tuberculose, Direction Provinciale de Kinshasa, Kinshasa, République Démocratique du Congo; 3 Programme National de Lutte contre la Tuberculose, Direction Nationale, Kinshasa, République Démocratique du Congo; 4 Damien Foundation Bangladesh, Dhaka, Bangladesh; 5 Institute of Tropical Medicine, Unit of Mycobacteriology, Department of Biomedical Sciences, 2000 Antwerp, Belgium; 6 Institute of Tropical Medicine, Unit of HIV and TB, Department of Clinical Sciences, 2000 Antwerp, Belgium

## Abstract

**Background:**

The incidence of acquired rifampicin resistance (RIF-ADR; RR) during first-line treatment varies.

**Objectives:**

Compare clinically significant RIF-ADR versus primary and reinfection RR, between regimens (daily versus no rifampicin in the continuation phase; daily versus intermittent rifampicin in the continuation phase) and between rural Bangladesh and Kinshasa, Democratic Republic of Congo.

**Methods:**

From patients with treatment failure, relapse, or lost to follow-up, both the outcome and baseline sputum sample were prospectively collected for *rpoB* sequencing to determine whether RR was present in both samples (primary RR) or only at outcome (RIF-ADR or reinfection RR).

**Results:**

The most frequent cause of RR at outcome was primary RR (62.9%; 190/302). RIF-ADR was more frequent with the use of rifampicin throughout versus only in the intensive phase (difference: 3.1%; 95% CI: 0.2–6.0). The RIF-ADR rate was higher with intermittent versus daily rifampicin in the continuation phase (difference: 3.9%; 95% CI: 0.4–7.5). RIF-ADR after rifampicin-throughout treatment was higher when resistance to isoniazid was also found compared with isoniazid-susceptible TB. The estimated RIF-ADR rate was 0.5 per 1000 with daily rifampicin during the entire treatment. Reinfection RR was more frequent in Kinshasa than in Bangladesh (difference: 51.0%; 95% CI: 34.9–67.2).

**Conclusions:**

RR is less frequently created when rifampicin is used only during the intensive phase. Under control programme conditions, the RIF-ADR rate for the WHO 6 month rifampicin daily regimen was as low as in affluent settings. For RR-TB control, first-line regimens should be sturdy with optimal rifampicin protection. RIF-ADR prevention is most needed where isoniazid-polyresistance is high, (re)infection control where crowding is extreme.

## Introduction

The term ‘acquired drug resistance’ (ADR) has long been erroneously used for any resistance observed in isolates from patients with recurrent TB. Resistance detected in patients with recurrence (bacteriological failure or relapse after completion) mainly represents primary resistance, already present at the start of treatment and causing recurrence.^1^

We define ADR as any new drug resistance reported by the laboratory during treatment monitoring, while also leading to bacteriologically and clinically overt recurrence. Newly appearing resistance without clinical correlate is often found to be temporary and for that reason has been called ‘transient’ in the British Medical Research Council (BMRC) studies.^2^ Transient resistance is thought to represent a condensate of remaining resistant mutants that were present at baseline and which will eventually be eradicated by the other drugs or host defence mechanisms, without any impact on treatment outcome. Determination of ADR requires drug susceptibility testing (DST) of the baseline and the recurrence strain at the end or after treatment with a regimen containing the drug studied. ADR must also be differentiated from reinfection with another strain. The pre- and post-treatment bacilli must be shown to be genetically identical by molecular fingerprinting techniques. Also laboratory or administrative errors (e.g. misidentification during sample collection or processing in the laboratory) may cause confusion.

ADR is a result of the selection under drug pressure of resistant mutants. The risk of ADR depends on the drug/target-specific frequency of naturally occurring resistant mutants selected under pressure of this drug, besides the initial bacillary load. It will thus be highest among microscopy-positive pulmonary patients. These are also the patients generating 5–10 times more secondary cases, as shown in older contact screenings as well as recent molecular epidemiological studies.^3,4^

A too low drug dosage with a too low concentration at the infection site (which may vary between and within patients) and poor adherence (particularly repeated periods of irregular intake and being on and off the drug) are generally seen as the common causes of ADR.^5,6^ Still, at population level these factors may have less impact than a lack of careful implementation of appropriate standard regimens for mass treatment by national treatment programmes (NTPs). Considering the diversity and frequency of problems NTPs and their patients are faced with, regimens should not only reach close to 100% treatment success in clinical trials, but also be highly successful under difficult field conditions. ADR may then very well be the most discriminating indicator, separating regimens that in a given population will ultimately lead to control of TB, from those likely to fail.

To maximally prevent ADR, the bactericidal, sterilizing and resistance-preventing effect of a treatment regimen must be optimal and provide for a margin of error (regimens must be ‘sturdy’). With sturdy, well implemented treatment regimens, almost no resistance is selected by and to the core drug, the regimen’s key drug, with high bactericidal and sterilizing effect.^7,8^

Although ADR is not necessarily limited to the core drug, we studied only resistance acquired to rifampicin (RIF-ADR). Complemented by genetic fingerprinting information, frequencies of molecularly defined RIF-ADR versus other types of rifampicin resistance (RR), such as primary and reinfection RR, were compared between regimens and settings. Patient cohorts (specified in Table 1) covered the last years of routine use of the 8 month 2(3)EHRZ/6HE and the following years with the 2(3)EHRZ/4HR regimen in Kinshasa Province of the Democratic Republic of Congo NTP (DRC) (H: isoniazid; R: rifampicin; E: ethambutol; Z: pyrazinamide). Likewise, cohorts enrolled in the Damien Foundation (DF) Bangladesh project comprised the last years of 2(3)EHRZ/6HT, the following years with 2(3)EHRZ/4H_3_R_3_ (thrice-weekly intermittent dosing) and the first years with the current 2(3)EHRZ/4HR regimen (T: thioacetazone). Our study is exceptional as it contains a very large number of failure and relapse cases, as well as patients lost to follow-up (LTFU) during their initial first-line treatment episode, presenting again at the health facilities.

**Table 1.** Genotypic rifampicin drug susceptibility test results for paired baseline and recurrence/LTFU samples, by first-line regimen and setting

Sample period and regimens	Total		DF Bangladesh						NTP DRC, Kinshasa Province			
			2002–03		2004–07		2009–11		2005–06		2007–09	
			2(3)EHRZ/ 6HT		2(3)EHRZ/4H_3_R_3_		2(3)EHRZ/4HR		2(3)EHRZ/6HE		2(3)EHRZ/4HR	
	*N*	(%)	*N*	(%)	*N*	(%)	*N*	(%)	*N*	(%)	*N*	(%)
*rpoB* genotypic DST results for pairs	1284		58		521		339		175		191	
RS/RS	913	(71.1)	48	(82.8)	391	(75.0)	266	(78.5)	116	(66.3)	92	(48.2)
RS/RR	107	(8.3)	1	(1.7)	54	(10.4)	18	(5.3)	7	(4.0)	27	(14.1)
RR/RR	195	(15.2)	3	(5.2)	61	(11.7)	28	(8.3)	36	(20.6)	67	(35.1)
RR/RS	9	(0.7)	1	(1.7)	1	(0.2)	3	(0.9)	1	(0.6)	3	(1.6)
NTM (one or both)	60	(4.7)	5	(8.6)	14	(2.7)	24	(7.1)	15	(8.6)	2	(1.0)
Total patients at RIF-ADR risk (RS baseline)	1020		49		445		284		123		119	
RS/RS	860	(84.3)	48	(98.0)	387	(87.0)	266	(93.7)	89	(72.4)	70	(58.8)
RS/RR, RIF-ADR	53	(5.2)	1	(2.0)	35	(7.9)	12	(4.2)	1	(0.8)	4	(3.4)
RS/RS, reinfection	54	(5.3)	0	(0)	4	(0.9)	0	(0)	27	(22.0)	22	(18.5)
RS/RR, reinfection	53	(5.2)	0	(0)	19	(4.3)	6	(2.1)	6	(4.9)	23	(19.3)
Total patients not at RIF-ADR risk (RR baseline)	204		4		62		31		37		70	
RR/RR, primary RR	190	(93.1)	3	(75.0)	61	(98.4)	26	(83.9)	35	(94.6)	65	(92.9)
RR/RR, reinfection	5	(2.5)	0	(0)	0	(0)	2	(6.5)	1	(2.7)	2	(2.9)
RR/RS, reinfection	9	(4.4)	1	(25.0)	1	(1.6)	3	(9.7)	1	(2.7)	3	(4.3)

E, ethambutol; H, isoniazid; R, rifampicin; Z, pyrazinamide; T, thioacetazone.

Reinfection was considered when fingerprinting showed a different strain in the recurrence/LTFU sample compared with the baseline one, or when the *rpoB* mutation differed. Regimens are written separating intensive and continuation phase by a forward slash (/). The numbers preceding a phase indicate its duration in months, for the intensive phase first the intended standard number of months followed between brackets by the number of months for non-conversion on microscopy for AFB at the intended end. Numbers in subscript following a drug symbol indicate intermittent treatment (in these cases spaced out to three times per week). By convention, a subscript number is omitted for daily treatment.

## Methods

Our study used prospectively collected routine sputa and data from the DF Bangladesh project, respectively the Kinshasa Province of the DRC NTP. Both implemented the programme on behalf of the NTP in a population of about 10 (Kinshasa), respectively 20 million (Bangladesh), with support of DF Belgium.^9,10^ Each project registered close to 10–000 pulmonary TB patients positive for acid-fast bacilli (AFB) annually. HIV, a known risk factor for acquired mono-rifampicin resistance,^11^ was virtually absent in the Bangladesh patients and of low level (about 6% HIV coinfection) in Kinshasa.^12^

Treatment outcomes (cure and recurrence/LTFU) were AFB-microscopy defined. For new cases and recurrences alike, the cut-offs were 1 AFB/100 high-power fields for Ziehl-Neelsen brightfield, and 5 AFB with auramine staining for LED fluorescence microscopy (widely used from 2009 onwards in DF Bangladesh) to declare a positive result.

TB work was integrated in general health services. Only Kinshasa had a TB sanatorium, mainly performing diagnosis followed by referral to clinics all over the metropolis. In Bangladesh, all (around 100) permanent DF diagnostic clinics participated, with DF-employed routine paramedical staff implementing the study. In Kinshasa, the Government staff of the 30 largest TB diagnostic and treatment centres implemented the study. Over the years, different NTP standard regimens were used successively as WHO recommendations changed, each defining a study arm (Table 1).

**Figure 1.**
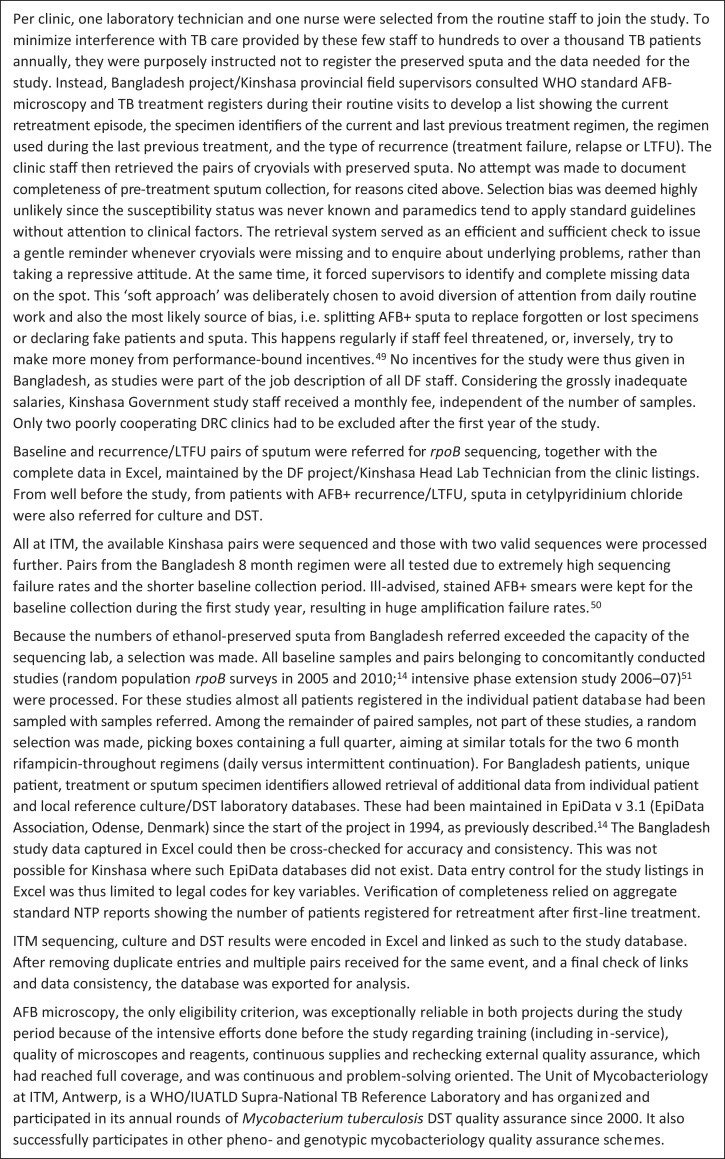
Data collection and sampling procedures.

All successively registered AFB+ cases were eligible, with a single study intervention, i.e. preservation of 0.5 mL diagnostic (new or recurrence) sputum added to 1 mL denatured ethanol in hermetically closing cryovials.^13^ To identify specimens unequivocally, transparent Scotch tape covered cryovial labels with laser-preprinted specimen-unique identification codes routinely used already on request forms, registers and treatment cards. Cryovials were kept at the clinics at room temperature in special collection boxes, arranged sequentially (code-defined) for easy retrieval in case of recurrence/LTFU. Baseline and recurrence/LTFU pairs of sputum were referred for *rpoB* sequencing. Procedures used for data collection and the selection of samples are shown in Figure 1.

The Supra-National TB Reference Laboratory at the Institute for Tropical Medicine (ITM) in Antwerp, Belgium, assured advanced testing such as DNA sequencing and fingerprinting, besides routine surveillance of main drug resistance.^14^ Sanger sequencing used extended primers covering the 81 bp rifampicin-resistance determining region (RRDR) as well as the 170 and 491 hotspots. Any non-silent mutation in these hotspots and adjacent mutations at codons 454 and 455 showing borderline MICs was considered to confer RR.^15^ Heteroresistance was determined by visual inspection for wildtype and mutant alleles double peaks, with a limit of detection (LOD) of around 25% minority mutant population.^16^ Fingerprinting using 24-loci MIRU-VNTR^17,18^ was performed systematically for pairs with WT *rpoB* (rifampicin susceptible, RS) before but RR after treatment (RS/RR pairs), for the reverse (RR/RS pairs), and for Kinshasa only a random selection of RR/RR and RS/RS pairs.

RIF-ADR was defined as an RS/RR pair with identical fingerprint, detected in the sputum from patients with AFB+ recurrence/LTFU. Fingerprinting was lacking for six patients with RS/RR. These events were counted as RIF-ADR, since 8/9 represented failure cases from Bangladesh, where reinfection was rare (see Results). Only patients with WT *rpoB* (RS) at start of treatment constituted the population at risk of RIF-ADR. Although misidentification could never be excluded, reinfection was considered when fingerprinting showed >1 allele difference in one or more MIRU loci between the recurrence/LTFU and baseline sample, or when the *rpoB* mutation differed. Patients with at least one non-TB mycobacterium (NTM) or RR/RR profile were excluded from the calculation of the frequency of RIF-ADR analysis, as were reversals (RR/RS pairs). Because of the small number of 2(3)EHRZ/6HT recurrences documented (*n *= 111), this cohort was merged with the other 8 month regimen with rifampicin in the intensive phase only [2(3)EHZR/6HE] to calculate RIF-ADR rates by regimen.

Stata’s (version 16.1, StataCorp LLC, College Station, TX, USA) test of proportions was used to calculate a 95% CI around the difference between two groups. The χ^2^ test was used to test whether there was an association between categorical variables.

The study was approved by the ITM Institutional Review Board (1233/18), including a waiver of the obligation to seek informed consent.

## Results

Table 1 shows *rpoB* genotypic DST results on sputum pairs, by regimen and by setting. Data were obtained for 1284 pairs, 918 (71.5%) from Bangladesh and 366 (28.5%) from Kinshasa (Table 1).

Of 1284, 60 (4.7%) showed NTM on either the baseline or recurrence/LTFU sample. Overall, and also in each setting, the most frequent cause of observed RR in the recurrence/LTFU sample was primary RR [overall: 190 of 302 (62.9%) with RR in the recurrence/LTFU sample].

Overall, in 54 (50.5%) of 107 RS/RR patients proof of reinfection was found. RS/RR reinfection was more frequent in Kinshasa (85.3%, 29/34) than in Bangladesh (34.3%, 25/73; difference: 51.0%, 95% CI: 34.9–67.2).

Overall, 53 RIF-ADR cases were identified. The RIF-ADR rate was higher for regimens with daily versus no rifampicin in the continuation phase [4.6% (16/352) versus 1.4% (2/139); difference: 3.1%, 95% CI: 0.2–6.0; excluding reinfections from the denominator] (Table 1). The difference was not significant in the separate settings. In Bangladesh, the RIF-ADR rate was higher for a regimen with intermittent than daily rifampicin in the continuation phase [8.3% (35/422) versus 4.3% (12/278); difference: 3.9%, 95% CI: 0.4–7.5; excluding reinfections from the denominator]. In a sensitivity analysis, with reinfections in the denominator, similar differences were found.

In baseline samples with the same recurrence/LTFU strain, of four patients with heteroresistant primary RR, none had heteroresistance at recurrence. In recurrence/LTFU samples after treatment with either daily or intermittent rifampicin in the continuation phase, heteroresistance was more frequent in patients with RIF-ADR (19.6%, 10/51) or RR reinfection (12.5%, 6/48), than in those with primary RR (0%, 0/152; χ^2^ test: *P *< 0.001).

Table 2 shows that RIF-ADR was more frequent after treatment failure than relapse/LTFU, particularly when rifampicin was used intermittently in the continuation phase [16.5% (28/170) versus 2.8% (7/252); difference: 13.7%, 95% CI: 7.8–19.6; excluding reinfections from the denominator].

**Table 2.** Genotypic rifampicin drug susceptibility test results for paired baseline and outcome samples, for initially rifampicin-susceptible TB in Kinshasa and Bangladesh, by first-line regimen and type of recurrence

Pair profiles	2(3)EHRZ/6HT or HE		2(3)EHRZ/4H_3_R_3_		2(3)EHRZ/4HR	
	*N*	(%)	*N*	(%)	*N*	(%)
Recurrence type: failure						
Total	69		185		313	
RS/RS	57	(82.6)	142	(76.8)	260	(83.1)
RS/RR (RIF-ADR)	2	(2.9)	28	(15.1)	14	(4.5)
RS/RR reinfection	2	(2.9)	13	(7.0)	24	(7.7)
RS/RS reinfection	8	(11.6)	2	(1.1)	15	(4.8)
Recurrence type: relapse or LTFU						
Total	102		260		90	
RS/RS	79	(77.5)	245	(94.2)	76	(84.4)
RS/RR (RIF-ADR)	0	(0)	7	(2.7)	2	(2.2)
RS/RR reinfection	4	(3.9)	6	(2.3)	5	(5.6)
RS/RS reinfection	19	(18.6)	2	(0.8)	7	(7.8)

E, ethambutol; H, isoniazid; R, rifampicin; Z, pyrazinamide; T, thioacetazone.

Reinfection was considered when fingerprinting showed a different strain in the recurrence/LTFU sample compared with the baseline one, or when the *rpoB* mutation differed. Regimens are written separating intensive and continuation phase by a forward slash (/). The numbers preceding a phase indicate its duration in months, for the intensive phase first the intended standard number of months followed between brackets by the number of months for non-conversion on microscopy for AFB at the intended end. Numbers in subscript following a drug symbol indicate intermittent treatment (in these cases spaced out to three times per week). By convention, a subscript number is omitted for daily treatment.

RIF-ADR after a regimen with rifampicin in the continuation phase was more frequent when resistance to isoniazid (Hr) was also found at recurrence/LTFU, compared with isoniazid-susceptible TB (Table 3). This was true for patients treated with intermittent rifampicin in the continuation phase [40.3% (23/57) versus 1.4% (2/139); difference: 38.9%, 95% CI: 26.0–51.8] as well as patients treated with daily rifampicin in the continuation phase [61.5% (8/13) versus 0% (0/45); difference: 61.5%, 95% CI: 35.1–88.0; Table 3]. In 53.8% (21/39) of patients with isoniazid polyresistant TB (Hr-poly) RIF-ADR was detected. Of 21, 11 had a HRES resistance profile (resistance to isoniazid, rifampicin, ethambutol and streptomycin).

**Table 3.** Acquired rifampicin resistance for initially rifampicin-susceptible TB, by type of isoniazid resistance on phenotypic DST and by first line regimen

	2(3)EHRZ/6HT or 6HE				2(3)EHRZ/4H_3_R_3_				2(3)EHRZ/4HR			
	No RIF–ADR		RIF–ADR		No RIF–ADR		RIF–ADR		No RIF–ADR		RIF–ADR	
	*N*	(%)	*N*	(%)	*N*	(%)	*N*	(%)	*N*	(%)	*N*	(%)
Total	137		2		387		35		336		16	
Hs	35	25.5	0	(0)	137	(35.4)	2	(5.7)	45	(13.4)	0	(0)
Any Hr	36	26.3	0	(0)	34	(8.8)	23	(65.7)	5	(1.5)	8	(50.0)
Of which H-mono	14	10.2	0	(0)	20	(5.2)	7	(20.0)	1	(0.3)	3	(18.8)
Of which H-poly	22	16.1	0	(0)	14	(3.6)	16	(45.7)	4	(1.2)	5	(31.3)
Hr unknown	66	48.2	2	(100)	216	(55.8)	10	(28.6)	286	(85.1)	8	(50.0)

E, ethambutol; H, isoniazid; R, rifampicin; Z, pyrazinamide; T, thioacetazone; Hs, isoniazid susceptible; Hr, isoniazid resistance; H-mono, isoniazid monoresistance; H-poly, isoniazid polyresistance.

Regimens are written separating intensive and continuation phase by a forward slash (/). The numbers preceding a phase indicate its duration in months, for the intensive phase first the intended standard number of months followed between brackets by the number of months for non-conversion on microscopy for AFB at the intended end. Numbers in subscript following a drug symbol indicate intermittent treatment (in these cases spaced out to three times per week). By convention, a subscript number is omitted for daily treatment.

## Discussion

Prevention of acquired core drug resistance was very high on the TB agenda when only one core drug was available, rifampicin.^19^ For the late Prof. Mitchison, bacteriologist of the BMRC clinical trials, which were instrumental in the development of modern TB chemotherapy, the first aim of chemotherapy was not cure but avoiding creation of ADR.^19^ Increasing levels of ADR and transmission of resistant strains, and thus increase in primary resistance, render standardized regimens ineffective for TB control. It has been shown that weak TB programmes can in the long term be worse than none at all. A rising proportion of resistant, sometimes chronic cases, may actually worsen the overall TB burden, even when reported cure rates for first-line treatment are improving.^20^ In a few decades since the introduction of rifampicin-throughout regimens (rifampicin in both the intensive and continuation phase), RR-TB became a threat for worldwide TB control,^21^ largely because of excessive emphasis on case detection and short-term treatment success, neglecting avoidance of core drug acquired resistance. This requirement for maximum protection, opting for sturdy regimens with sufficient margin for error seems no longer a concern. At present ADR is largely ignored in drug or regimen evaluations, WHO recommendations and guidelines.^22,23^ The failure outcome definition, in fact a composite outcome including both bacteriological and safety adverse outcomes, even hides the main alert parameter, true bacteriological failure.^24^

The 8 month regimen without rifampicin in the continuation phase was purposely selected from the diversity of BMRC trial-proven regimens^25^ by the late Dr Styblo of the International Union against TB and Lung Disease (IUATLD) for NTPs piloting short-course chemotherapy in difficult settings. Since RR was untreatable at that time (around 1980), particularly in low-income settings, he rightly prioritized prevention of RIF-ADR over maximum short-term success. He showed that the results obtained in clinical trials could be achieved under difficult conditions in several low-income countries: cure rates increased by some 20% while RR stayed at the same very low level after decades of country-wide implementation.^26,27^

Our study showed that in both Bangladesh and the DRC, RIF-ADR became significantly more frequent after changing from 8 month regimens, which did not contain rifampicin in the continuation phase, to 6 month regimens with daily rifampicin throughout (increase from 1.4% to 4.6% at recurrence/LTFU, similar for both settings). While RIF-ADR became more frequent, annual reports of DF Bangladesh as well as Kinshasa Province showed a consistent modest increase of treatment success from 1%–2% below 90% to 1%–2% above 90% as the 6 month rifampicin-throughout regimen was implemented. In the same period Cat2 (retreatment regimen, 8 month rifampicin-throughout) outcomes worsened, as a higher proportion had RR when starting the Cat2 treatment regimen. The Cat2 regimen was designed to overcome isoniazid-resistant, rifampicin-susceptible (HrRs-TB) after an unsuccessful first treatment with an isoniazid-throughout regimen using rifampicin only in the intensive phase.^14^

When in DF Bangladesh during some years the regimen with thrice-weekly HR in the continuation phase was used, the RIF-ADR was even as high as 7.9%, which increased the RR prevalence from well below 1% to around 2%. It decreased again after the change to the rifampicin daily regimen and concomitant improved detection and treatment of RR.^14,28^ Direct observation of drug intake (DOT) can improve adherence and contribute to the prevention of RIF-ADR. But, at least in low-income settings, good supportive DOT is truly rare, and even the best DOT is useless with poorly conceived standard regimens. To make DOT more feasible by changing to intermittent dosing of standard regimens for mass application in poor populations is likely to be a capital error. HR thrice-weekly (or even worse, twice-weekly), baseline HrRs-TB and/or rapid isoniazid acetylators are each more permissive to RIF-ADR. Combined, these factors caused extremely high rates of failure and RIF-ADR in South India.^29^ When NTPs had to switch to rifampicin throughout, only India was allowed to use intermittent thrice-weekly dosing throughout treatment. Nowadays, India has the largest burden of RR-TB worldwide, with around 3% RR in new cases compared with below 1% in neighbouring Bangladesh.^30^ Peru’s highly reputed NTP has for years used a twice weekly continuation phase for first-line (Cat1) and retreatment (Cat2) regimens.^31^ Together with unusually intense transmission^32^ this practice may explain the rise of primary RR to 6%–7%. Regimens often show excellent results in clinical trials under ideal conditions, but mass application in the field may yield disastrous results. The equivalence of intermittent with daily HR dosing in various clinical trials^2^ contrasts with the high frequency of relapse, failure and RIF-ADR in routine care.^33,34^ The frequency of bacteriologically adverse outcomes has been shown to be proportional with the spacing of doses and/or due to non-extension of the intensive phase for patients with a high bacillary load.^18^

A regimen is sturdy when it shows strong ADR-preventing activity to its core drug, the most powerful drug of the regimen.^7^ In the 6 month regimens, isoniazid protects rifampicin. In the old, very long first-line regimens without rifampicin, isoniazid acted as an initially extremely bactericidal drug, but imperfect because poorly sterilizing.^25^ Also in the current study the presence of resistance to isoniazid was strongly associated with RIF-ADR, also shown in our previous publication.^35^ However, since only recurrences were included in the present study, we could not assess the proportion of baseline HrRs ending with RIF-ADR. We and others have shown that H-poly (with resistance to both ethambutol and streptomycin; HrRsErSr) results in poor outcomes, while recurrence due to H-mono is relatively rare.^35,36^ HrSr resistance was by far the most frequent type of H-poly at recurrence in our study, even without inclusion of streptomycin in the regimen, but half of RIF-ADR showed the HrRrErSr profile on phenotypic DST. Ethambutol is added in the first-line regimen to assure ‘back-up’ resistance preventing activity in case of initial HrRs.^7^ Ivory Coast, which used 2HRZ/4HR without ethambutol and without DOT, was the first African country where over 5% of primary RR among new patients was found.^37^ The original first-line 8 month regimen used the—in clinical trials—more protective streptomycin,^25^ until all-oral ethambutol-containing treatment was preferred mainly to avoid HIV-related toxicity. Early resistance preventing activity was sacrificed, as happens again these days for RR-TB regimens with amikacin, judged less important than serious drug adverse events and painful injections.^7,38,39^

Our findings show that the presently used first-line regimen does not provide sufficient protection in case of H-poly, or even H-mono. In the DF Bangladesh population, Hr-related RIF-ADR percentages and risks are worse for the daily HR regimen, but numbers show the reverse. The intermittent regimen allowed far more recurrences/LTFU from Hr with more ADR (57 Hr/422 recurrences/LTFU, 23 with RIF-ADR) than the daily regimen (13 Hr/352, 8 with RIF-ADR). Prevalences of baseline Hr measured in five random surveys between 1995 and 2015, and thus across all regimens analysed, had remained unchanged.^14^ With baseline Hr, the intermittent continuation phase thus seemed to permit about three times more recurrences as well as RIF-ADR than daily throughout.

How the current first-line regimen should be strengthened or modified in settings with a high prevalence of H-poly should be studied. The same applies to settings with a high prevalence of both H-mono and HIV coinfection.^11^ Unfortunately DST to ethambutol and streptomycin is notoriously unreliable by any method, leaving the earliest possible detection of RIF-ADR as the only option for treatment response monitoring.^40^ Rapid, decentralized molecular DST, particularly Xpert MTB/RIF, has allowed a tremendous progress regarding the rapid detection of primary RR.^30^ Nevertheless, such tests may not so soon become accessible to all patients in need.^30^ Moreover, for the monitoring of treatment response they have proven too little specific.^41–42^ Amplification speed changes are slow, erratic and thus too uncertain as the sole or main diagnostic element. Together with clinical and AFB-microscopy findings, they should rather be considered as alerts triggering close follow-up. For the early detection of RR in patients with baseline RS-TB, Xpert MTB/RIF may have a role to play. However, results should always be interpreted together with the clinical presentation, to distinguish transient RR from RR causing clinical treatment failure.

In addition, because of fairly regularly missed primary RR—especially when using rapid phenotypic DST or molecular assays targeting only the 81 bp region^43^—and because of the increased risk for RIF-ADR, patients reported with H-poly are also at high risk to end as fluoroquinolone-resistant MDR if treated with the levofloxacin-strengthened first-line WHO regimen for HrRs-TB.^35^ For long-term TB control not creating ADR to core drugs should be prioritized over emphasis on immediate treatment success. Without this, shifting to new (core) drugs can’t bring lasting progress.^44^ Before a background regimen has been identified that limits ADR to a minimum in the average NTP, new TB core drugs should not become the standard of care for TB that is still susceptible to an older core drug. The ‘ancient’ vision of TB control, with emphasis on ADR prevention, is still highly relevant considering the high rates of ADR to bedaquiline in settings where it has been used for a sufficient time on a sufficiently large scale, despite background regimens respecting current recommendations which strongly emphasize using these newer drugs.^44,45^

How to interpret the level of RIF-ADR among those treated with the 6 month daily rifampicin regimen and at risk, thus with initially rifampicin-susceptible TB? The frequency of RIF-ADR was 3%–4%, very similar in both settings. Considering (i) about 5% recurrence cases during the study period,^35^ similar to what is shown in clinical trials;^46^ and (ii) that 2/3 of 5% recurrences were not at risk [67% (91/134) showed baseline RR, and were excluded from this calculation], around 0.5 per 1000 (33% at risk × 5% recurrence × 4% with RIF-ADR) patients had RIF-ADR. This estimate is similar to the 1/1000 reported from affluent countries.^47^

The main strength of our study was the prospective data collection with direct sequencing from the sputum specimen, necessitating large scale storage of baseline sputa to capture a relatively rare event. Bacteriology both at the sites and the reference laboratory was of documented high quality and all recording consistent over the years. Our study had some limitations. First, because of its integration in NTP routine (also a strength), randomization to regimens was impossible as cohorts were enrolled successively. Some reinfection interpretation may have been confused with sample identification switch. However, the proportion with reinfection varied. The higher frequency of reinfection when RR reinfection truly is an advantage for progressive TB [in patients with RS/RR and on rifampicin treatment, compared with patients with baseline resistance (RR/RR)], suggests that indeed reinfection was mostly real. Among patients at risk and treated with a rifampicin daily regimen, RR reinfection was much more frequent than RIF-ADR in Kinshasa (19.3% against 3.4%), compared with Bangladesh (2.1% against 4.2%). The settings are very different. While extreme crowding in the Kinshasa suburbs and waiting areas of extremely busy health facilities contribute to reinfection,^48^ the setting in Bangladesh is rural with more open space in health centres and administrative and engineering ventilation measures in DF hospital TB wards.

In conclusion, the 6 month rifampicin daily regimens create more RIF-ADR than the original DOT regimens with rifampicin during the intensive phase only. When rifampicin is used in the continuation phase, RIF-ADR is more frequent with intermittent dosing. Still RIF-ADR is rare with rifampicin daily throughout, also in difficult settings, comparable with its frequency in high-income countries. However, the background regimen has to be sturdy for the intended population and setting. An efficient strategy for the earliest possible RIF-ADR detection would be far more effective than current, often misleading or too late, end-of-intensive-phase AFB smears. The alternative, identification at baseline of H-poly cases, who are at high risk of RIF-ADR, is not (reliably) possible given the limitations of ethambutol and streptomycin DST. In crowded settings, infection control measures are key to stop RR-TB from spreading to other patients. Elsewhere, sturdy first-line regimens together with early RR detection and effective RR-TB treatment will allow performant NTPs to keep acquisition as well as transmission of RR-TB at a minimum, with good prospects for TB control irrespective of resistance to other first-line drugs.
